# Mining distinct aldehyde dehydrogenase 1 (ALDH1) isoenzymes in gastric cancer

**DOI:** 10.18632/oncotarget.8294

**Published:** 2016-03-23

**Authors:** Jia-Xin Shen, Jing Liu, Guan-Wu Li, Yi-Teng Huang, Hua-Tao Wu

**Affiliations:** ^1^ Department of General Surgery, The First Affiliated Hospital of Shantou University Medical College, Shantou, PR China; ^2^ Chang Jiang Scholar's Laboratory, Shantou University Medical College, Shantou, PR China; ^3^ Guangdong Provincial Key Laboratory for Diagnosis and Treatment of Breast Cancer, Cancer Hospital of Shantou University Medical College, Shantou, PR China; ^4^ Open Laboratory for Tumor Molecular Biology/Department of Biochemistry, The Key Laboratory of Molecular Biology for High Cancer Incidence Coastal Chaoshan Area, Shantou University Medical College, Shantou, PR China; ^5^ Health Care Center, The First Affiliated Hospital of Shantou University Medical College, Shantou, PR China

**Keywords:** ALDH1, gastric cancer, prognosis, KM plotter, hazard ratio (HR)

## Abstract

Aldehyde dehydrogenase 1 (ALDH1) consists of a family of intracellular enzymes, highly expressed in stem cells populations of leukemia and some solid tumors. Up to now, 6 isoforms of ALDH1 have been reported. However, the expression patterns and the identity of ALDH1 isoenzymes contributing to ALDH1 activity, as well as the prognostic values of ALDH1 isoenzymes in cancers all remain to be elucidated. Here, we studied the expressions of ALDH1 transcripts in gastric cancer (GC) compared with the normal controls using the *ONCOMINE* database. Through the *Kaplan-Meier plotter* database, which contains updated gene expression data and survival information of 876 GC patients, we also investigated the prognostic values of ALDH1 isoenzymes in GC patients. It was found that when compared with normal tissues, ALDH1A1 mRNA expression was downregulated, whereas ALDH1A3 and ALDH1B1 were upregulated in GC patients. In survival analyses, high ALDH1A1 and ALDH1B1 expressions were associated with better overall survival (OS) in all GC patients. In addition, high transcription activity of ALDH1A1 predicted better OS in gastric intestinal type adenocarcinoma, but not in diffuse gastric adenocarcinoma. GC patients with high mRNA level of ALDH1B1 showed better OS in gastric intestinal type, and worse OS in diffuse type. Oppositely, high transcription activities of ALDH1A2, ALDH1A3 and ALDH1L1 predicted worsen overall survival in GC patients, suggesting that these isoenzymes might be responsible mainly for the ALDH1 activities in GC. These data provides ALDH1A2, ALDH1A3 and ALDH1L1 as excellent potential targets for individualized treatment of GC patients.

## INTRODUCTION

Gastric cancer (GC), one of the serious threats to human health contributing to a malignant tumor in the digestive system, is currently the third most common cause of deaths associated with cancer worldwide, accounting for approximately 9% of cancer-related deaths [1]. Although survival advantages can be achieved by surgical resection, combined with adjuvant/post-operative chemotherapy and/or radiotherapy in patients with early stage GC, almost two-thirds GC patients suffer from locally advanced or metastatic diseases, with poor survival outcomes of a median of 10 months period [2]. However, only a handful of new chemotherapeutic agents or molecular-targeted therapies have been developed. Therefore, further investigations in the underlining mechanisms of incidence and progression, as well as determining the development of prognosis and treatment biomarkers, will provide a comprehensive picture of their impacts on survival and invaluable reference allowing new agents/therapies.

Recently, Brungs D *et al*. systematically reviewed the current evidence for cancer stem cell (CSC) in GC [3], which the evidence includes superior tumor initiation, growth, and metastatic potential to that of other tumor cells [4]. It is reported that CSC is also responsible for the renewal of tumor mass after systemic treatment and development of sub-clones with more resistance to chemotherapeutics [5]. So far, it has been a challenge to identify a CSC population within a tumor. The methods for CSC identification and isolation mainly depend on side population (SP) assay and combinations of cell surface markers expression [3]. Recently, many protein are reported to be the putative gastric cancer CSC markers, such as CD26 [6], CD44 [7], ALDH1 [8], and CD133 [9], and Lgr5, which was reported to be co-localized with other CSC markers and may be functionally-associated [10].

Aldehyde dehydrogenase (ALDH) is a family of intracellular enzymes that are involved in cellular detoxification, differentiation, and drug resistance by oxidation of cellular aldehydes [11]. ALDH1, a marker of CSC in a variety of cancers, plays as a modulator for cell proliferation and stem cell differentiation, as well as resistance to chemotherapeutic agents [12]. ALDH1 positivity in diffuse-type lymph node metastasis was significantly higher than that in primary, predicting that CSC markers are important in tumor invasion and metastasis and may be prognostic markers in GC patients [13]. However, it is found that the expression of ALDH1 and REG4 can be reduced by TGF-β signaling pathway, resulting in decreasing ALDH1+ cell population size and tumorigenic capacity of diffuse-type gastric carcinoma-initiating cells [14]. CSC markers, such as ALDH1, LGR5 and CD166 were expressed in very low levels in normal human gastric mucosa or young rat gastric mucosa, compared with in H. pylori gastritis and gastric adenocarcinoma as well as in normal gastric mucosa in aged rats [15].

Although ALDH1 has been identified as a reliable marker of GC and other solid tumors, the functions of different ALDH1 isoforms in contribution to ALDH1 activities and prognostic value are still unclear. Recently, Wu *et al*. and You *et al*. reported the distinct prognostic values of ALDH1 isoenzymes in breast cancer and non-small-cell lung cancer (NSCLC), respectively [16, 17], providing a comprehensive illustration of the prognostic value of individual ALDH1 isoenzymes. In this study, we extended the research field to gastric cancers, to determine the expression pattern of ALDH1 isoenzymes in gastric cancer *versus*. normal tissues and distinct prognostic value of them.

## RESULTS

### Different transcription levels of ALDH1 isoforms in gastric cancers

**Figure 1:**
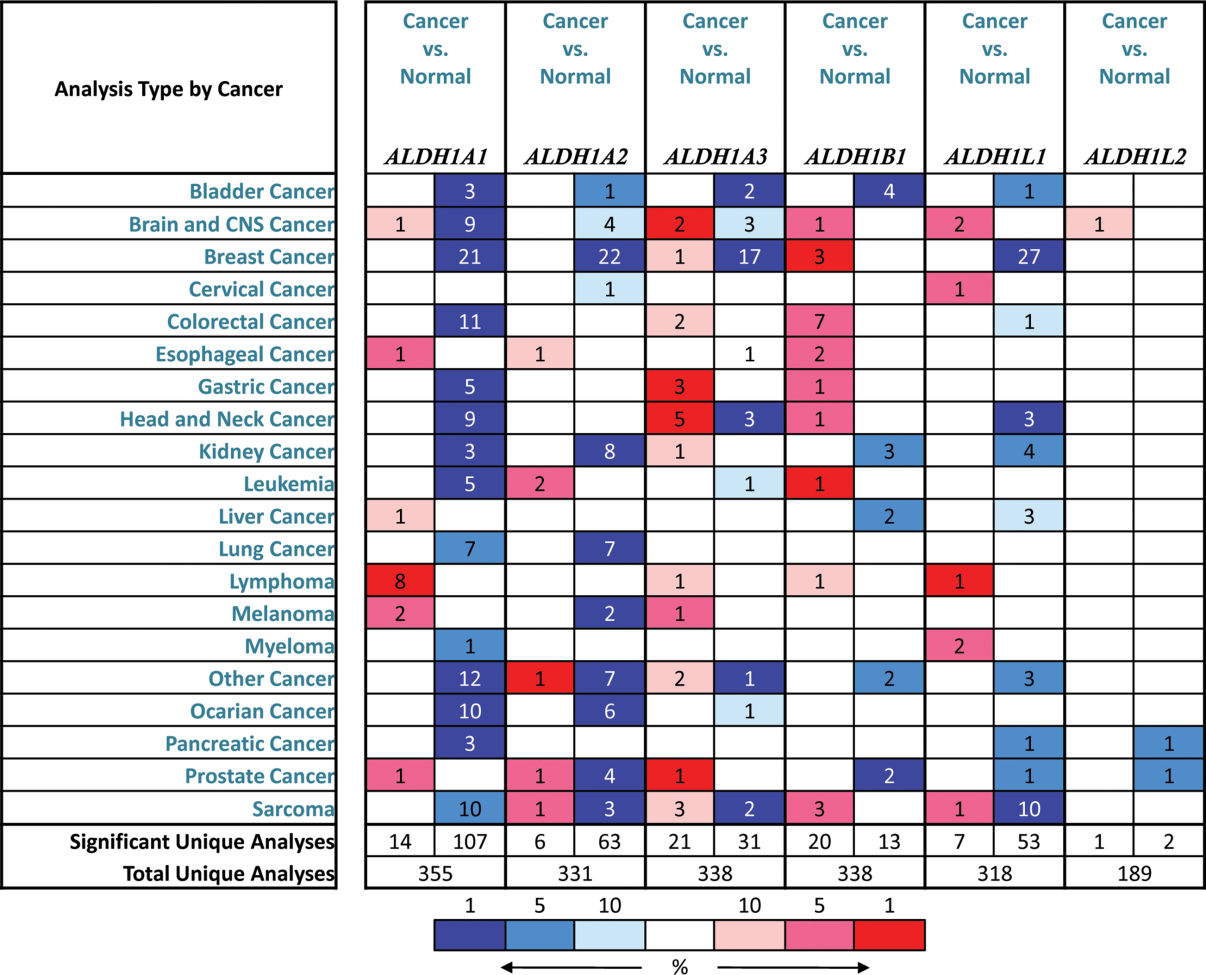
Different ALDH1 isoform mRNA expression in different tumor types This graphic showed the numbers of datasets with statistically significant mRNA overexpression (red) or downexpression (blue) of the target gene (cancer *vs*. normal tissue). The *p* value threshold is 0.01. The number in each cell represents the number of analyses that meet the threshold within those analysis and cancer types. The gene rank was analyzed by percentile of target gene in the top of all genes measured in each research. Cell color is determined by the best gene rank percentile for the analyses within the cell.

**Figure 2:**
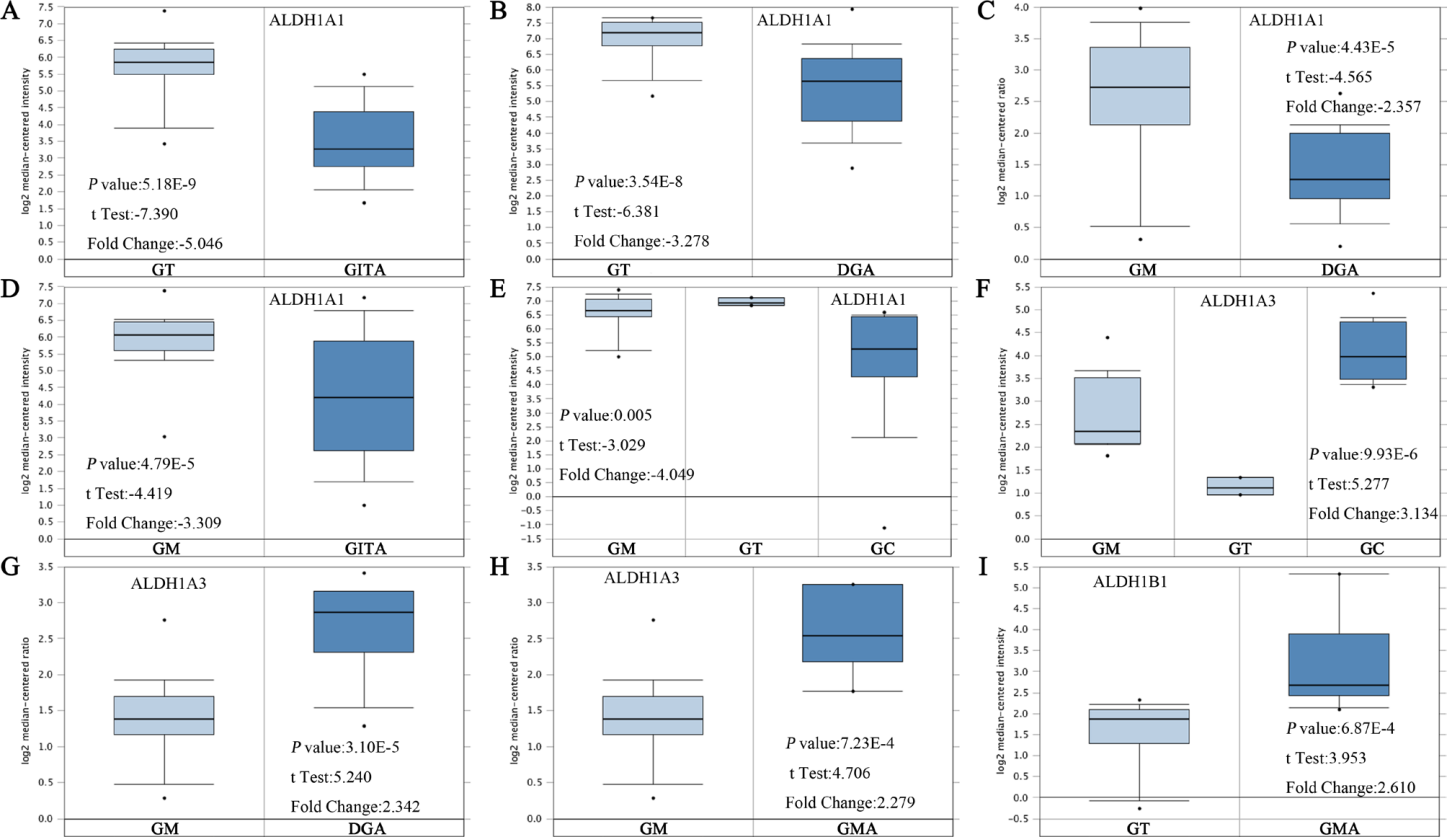
ALDH1 isoforms analysis in gastric cancer (*ONCOMINE* database) Box plots derived from gene expression data in *ONCOMINE* comparing expression of a specific ALDH1 isoform in normal and GC tissue. The *p* value was set up at 0.01 and fold change was defined as 2. (**A**–**E**) Comparison of ALDH1A1 mRNA expression. (**F**–**H**) Comparison of ALDH1A3 mRNA expression. (**I**) Comparison of ALDH1B1 mRNA expression [18–21]. Abbreviation: gastric tissue (GT), gastric intestinal type adenocarcinoma (GITA), diffuse gastric adenocarcinoma (DFA), gastric mucosa (GM), gastric cancer (GC), gastric mixed adenocarcinoma (GMA).

Up to now, 6 sub-members were reported of the ALDH1 family in Figure 1 [16]. *ONCOMINE* analysis of 6 ALDH1 sub-members in cancer *vursus*. normal samples showed that ALDH1A1 was significantly downregulated in different types of GC in different datasets. In Cho's dataset, the transcription levels of ALDH1A1 in both gastric intestinal type adenocarcinoma and diffuse gastric adenocarcinoma were lower than that in gastric tissues (Fold changes were −5.046 and −3.278, respectively) (Figure 2A and 2B) [18]. The ALDH1A1 mRNA level was also decreased in diffuse gastric adenocarcinoma (Figure 2C) and gastric intestinal type adenocarcinoma (Figure 2D), compared with gastric mucosa [19, 20]. In another dataset with only 27 samples, the ALDH1A1 mRNA level in GC was downregulated, compared with both gastric mucosa and gastric tissue (Figure 2E) [21].

In contrast, overexpressions of ALDH1A3 and ALDH1B1 were found in GC compared with normal tissues (Figure 1). In Wang's dataset, the ALDH1A3 mRNA level was significantly upregulated in GC, with 3.134 fold change (*p* = 9.93E-6) (Figure 2F) [21]. Consistently, in another dataset with 132 samples from patients with different types of gastric adenocarcinoma, the mRNA level of ALDH1A3 was over 2 up-fold change than in gastric mucosa (Figure 2G and 2H) [18]. However, only one dataset showed the significant fold change (2.610, *p* = 6.87E-4) of ALDH1B1 mRNA levels between cancer and normal tissue (Figure 2I).

### The prognostic value of different ALDH1 isoenzymes in gastric cancer

As previously indicated [16, 17], among all the six ALDH1 isoenzymes, the recently reported ALDH1L2 isoform by Krupenko NI *et al*., was not found in www.kmplot.com, was not recruited in the screening pool [22].

Survival curves were plotted in www.kmplot.com for all GC patients (*n* = 876), gastric intestinal type adenocarcinoma (*n* = 320), diffuse gastric adenocarcinoma (*n* = 241), HER2-negative GC (*n* = 532), and HER2-positive GC (*n* = 344). As the sample size was too small in patients with gastric mixed adenocarcinoma (*n* = 32), we did not analyze the survival curves in this group. We assessed the prognostic effect of the mRNA expression of ALDH1A1 (the Affymetrix IDs is 212224_at), ALDH1A2 (the Affymetrix IDs is 207016_s_at), ALDH1A3 (the Affymetrix IDs is 203180_at), ALDH1B1 (the Affymetrix IDs is 209646_×_at) and ALDH1L1 (the Affymetrix IDs is 205208_at) in www.kmplot.com.

**Figure 3:**
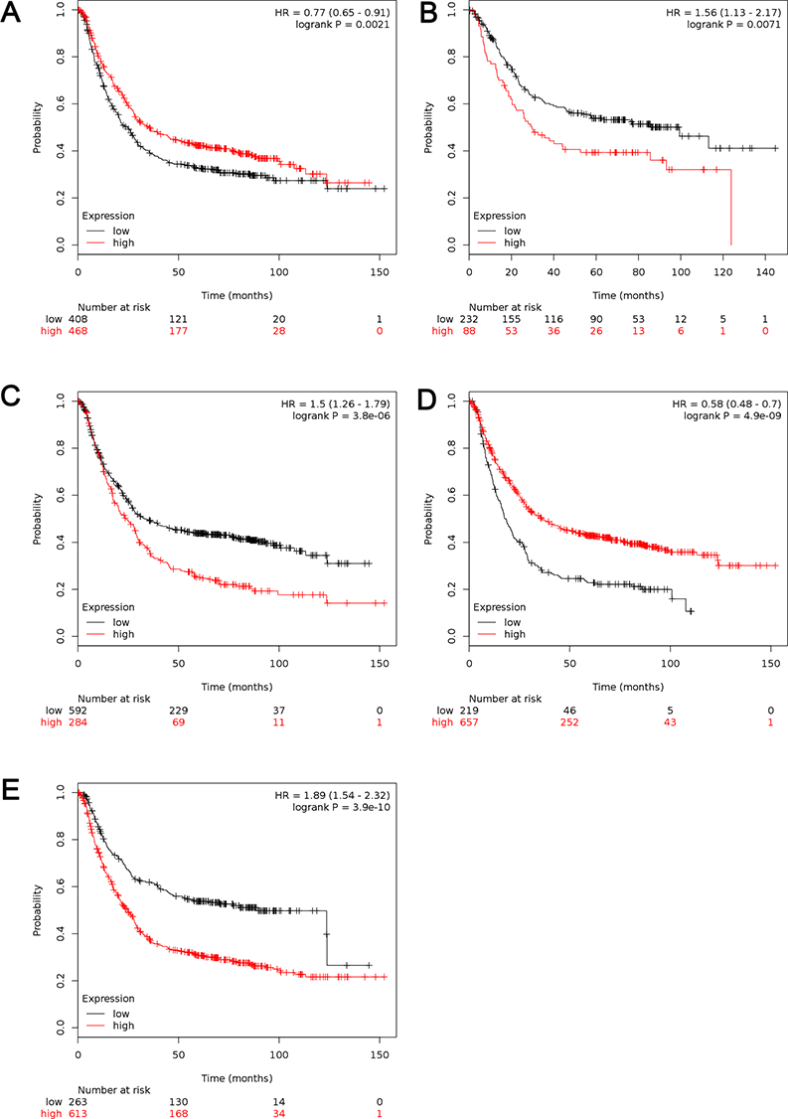
The prognostic value of mRNA level of ALDH1 isoenzymes in all GC patients (*n* = 876) (**A**) ALDH1A1 (212224_st) (**B**) ALDH1A2 (207016_s_at). (**C**) ALDH1A3 (203180_at). (**D**) ALDH1B1 (209646_×_at). (**E**) ALDH1L1 (205208_at). Data was analyzed using Kaplan-Meier plotter.

In Figure 3, increased mRNA levels of ALDH1A1 and ALDH1B1 expressions were associated with longer OS, *i.e.* better prognosis of GC patients, with HR = 0.77 (0.65–0.91), *p* = 0.0021 (Figure 3A) and HR = 0.58 (0.48–0.7), *p* = 0.0000 (Figure 3D), respectively. In contrast to other isoenzymes, high mRNA levels was found to be correlated to worsen OS in GC patients for ALDH1A2 (HR = 1.5 [1.22–1.85], *p* = 0.0001) (Figure 3B). Interestingly, high mRNA level of ALDH1A3 and ALDH1L1 were also significantly associated with worsen OS in all GC patients, with HR = 1.5 (1.26–1.79), *p* = 0.0000 (Figure 3C) and HR = 1.89 (1.59–2.32), *p* = 0.0000 (Figure 3E).

**Figure 4:**
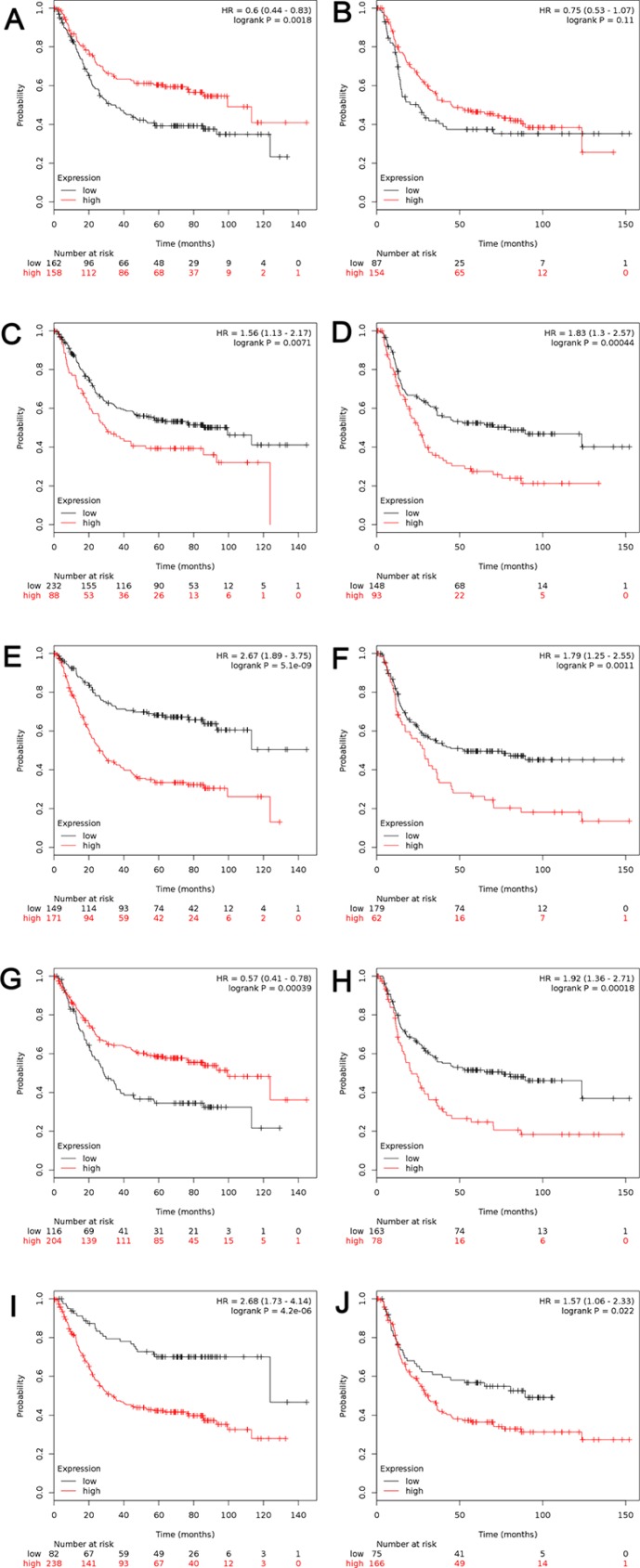
The prognostic value of mRNA level of ALDH1 isoenzymes in patients with gastric intestinal adenocarcinoma (*n* = 320) and diffuse gastric adenocarcinoma (*n* = 241) (**A**, **B**) ALDH1A1 (212224_st) (**C**, **D**) ALDH1A2 (207016_s_at). (**E**, **F**) ALDH1A3 (203180_at). (**G**, **H**) ALDH1B1 (209646_×_at). (**I**, **J**) ALDH1L1 (205208_at). (A, C, E, G, I) Survival curves in patients with gastric intestinal adenocarcinoma (*n* = 320). (B, D, F, H, J) Survival curves in patients with diffuse gastric adenocarcinoma (*n* = 241). Data was analyzed using Kaplan-Meier plotter.

For further analysis, the patients have been sub-grouped by their types of cancer. High transcriptional expression of ALDH1A1 was associated with longer OS in gastric intestinal type adenocarcinoma, with HR = 0.6 (0.44–0.83), *p* = 0.0018 (Figure 4A), but not in diffuse gastric adenocarcinoma (Figure 4B). As another good predictor, high level of ALDH1B1 predicted better OS in gastric intestinal type adenocarcinoma, with HR = 0.57 (0.41–0.78), *p* = 0.0000 (Figure 4G) and worse OS in diffuse gastric adenocarcinoma, with HR = 1.92 (1.46–2.51), *p* = 0.0000 (Figure 4H). Interestingly, for ALDH1A2 (Figure 4C and 4D), ALDH1A3 (Figure 4E and 4F) and ALDH1L1 (Figure 4I and 4J), high mRNA level was found to be significantly correlated to worsen OS in both gastric intestinal type adenocarcinoma and diffuse gastric adenocarcinoma, with the same trend as in all GC patients.

HER2 status in GC was reported as a potential target for individual therapy [23]. So, the survival curves have been stratified by HER2 status to determine who might benefit from targeted therapy (Table 1). Although exhibiting the similar expression trend, upregulated ALDH1A1 was significantly associated with better OS in HER2-negative GC, with HR = 0.7 (0.56–0.88), *p* = 0.0018, not in HER2-positive GC (Supplementary Figure S1A and S1B). Increased ALDH1B1 was only significantly correlated with better OS in HER2-positive GC, with HR = 0.55 (0.44–0.69), *p* = 0.0000, but not in HER2-negative GC (Supplementary Figure S1G and S1H). Interestingly, except ALDH1A1 and ALDH1B1, other three isoenzymes all predicted worse OS with high expression in both HER2-negative and HER2-positive GC (Table 1).

**Table 1:** The Hazard ratio (HR) of ALDH1 isoenzymes in HER2-negative and HER2-positive GC patients

	HER2-negative			HER2-positive		
	HR	95% CI	*P* value	HR	95% CI	*P* value
ALDH1A1	0.7	0.56–0.88	0.0018	0.89	0.69–1.16	0.4
ALDH1A2	1.47	1.18–1.84	0.0007	1.5	1.09–2.07	0.013
ALDH1A3	1.68	1.34–2.11	0.0000	1.6	1.23–2.07	0.0004
ALDH1B1	0.8	0.64–1.01	0.064	0.58	0.44–0.76	0.0000
ALDH1L1	1.92	1.46–2.51	0.0000	1.42	1.1–1.85	0.0072

HR: Hazard ratio.

CI: Confidence Interval.

## DISCUSSION

Gastric cancer (GC), mainly developing from the innermost lining of the stomach, presents the highest mortality rate among all digestive tract malignancies, mainly because of chemoradiotherapy resistance and distant metastasis [1]. It is crucial to illustrate the pathogenesis of GC, as well as to find novel prognostic strategies, early diagnostic tools and effective therapeutic approaches. On the other hand, strong evidences supported that CSC possess unique properties, such as uncontrolled growth and high tumorigenic and migration potential. CSCs are responsible for tumor initiation, progression, metastasis and chemoradiotherapy resistance. Among the identified CSC markers, ALDH1 is one found in a broad cancer spectrum. However, there are few reports available to analyze the expression of ALDH1 in GC, not to mention the different ALDH1 isoforms. In our study, we analyzed and discussed distinct roles of ALDH1 isoenzymes in prognostic value of GC.

ALDH1 mainly functions as a retinoic acid enzyme, catalyzing and conversing retinol to retinoic acid. As ALDH1A1 can inactivate integral chemotherapy agents, particularly cyclophosphamide, it is indicated that high ALDH1A1 expression in tumors may contribute to chemotherapy resistance of tumors [24]. Condello *et al*. recently reported that an ALDH1A1-specific inhibitor was used to block ovarian cancer cell proliferation and survival [25]. Recently, Li *et al*. reported that the overexpression of ALDH1A1 in protein level was correlated with poor OS and recurrence-free survival (RFS) in GC [26]. However, we found that the mRNA level of ALDH1A1 showed opposite prognostic value, *viz.* transcripts of ALDH1A1 were down-regulated in all types of GC and high mRNA expression of ALDH1A1 predicted better prognosis. Not surprisingly, Liu *et al*. concluded that high expression of ALDH1A1 transcripts may be an independent predictor of a favorable outcome in patients with triple-negative breast cancer, possibly due to the process of sample collection as suggested [27]. However, in our study, the datasets from different laboratories showed consistent results, indicating that the mRNA level of ALDH1A1 may be a good prognostic marker in GC, which could be opposite to their protein levels.

ALDH1B1, a mitochondrial ALDH, is another potential good prognostic marker in GC, sharing 65% homology in peptide sequence with cytosolic ALDH1A1 in human. ALH1B1 catalytically metabolizes a wide range of aldehyde substrates, including acetaldehyde and products of lipid peroxidation (LPO), and is activated in ethanol metabolism [28]. In colon cancer, ALDH1B1 is dramatically upregulated [29], and by up-regulating Wnt/β-Catenin, Notch and PI3K/Akt signaling pathways, ALDH1B1 can promote colon cancer tumorigenesis, providing a novel target to prevent or treat colon cancer [30]. The mRNA level of ALDH1B1 was higher in gastric mixed adenocarcinoma than in gastric tissues in Cho's research dataset [18]. However, in Kaplan-Meier plotter analysis, the ALDH1B1 in this study was found to be a good prognostic marker for OS in patients with all types of GC and intestinal type gastric adenocarcinoma, a poor prognostic marker for OS in patients with diffuse gastric adenocarcinoma. With the preliminary results, more research would be helpful to explore the mechanism of ALDH1B1 in GC.

ALDH1A2 was reported as a candidate tumor suppressor in prostate cancer, downregulated on early stage of human prostate cancer [31, 32]. Controversially, an *in vitro* study showed that K562 leukemia and H1299 lung cancer cell with ALDH1A2 overexpression exhibited higher cell proliferation rates, higher clonal efficiency, and increased drug resistance to 4-hydroperoxycyclophosphamide and doxorubicin [33]. In this study, we first reported that high ALDH1A2 transcription might predict poorer survival in GC patients, in which may involve the synthesis of retinoic acid pathway.

ALDH1A3 may promote progress and metastasis of various cancers, such as prostate cancer [34], gallbladder cancer [35], breast cancer [36], ovarian cancer [37] and NSCLC [38], as well as gastric cancer in our study, and be identified as the biomarkers for poor prognosis. ALDH1A3 responded to androgen dihydrotestosterone treatment and increased the oxidation of retinal to RA in human prostate cancer cells [34], and was supposed to be the predominant isoenzyme responsible for ALDH activity and tumorigenicity in most NSCLC [38]. As low expression of ALDH1A3 may be due to dysregulation of hypermethylation status of ALDH1A3 promoter [39] or suppression of miR-125a/b [40], the mechanism of ALDH1A3 in GC may involve differential retinoic acid signaling and hypermethylation of ALDH1A3 promoter, providing new therapy approaches.

ALDH1L1 (FDH, a folate metabolic enzyme with tumor suppressor-like properties) is cell-specifically expressed and involved in central nervous system development and reduced proliferation [41]. Interestingly, ALDH1L1 was reduced in clinically aggressive compared with Pilocytic [42]. ALDH1L1 could inhibit cancer cell motility via dephosphorylation of cofilin by PP1 and PP2A in folate-specific manner [43]. In hepatocellular carcinoma, decreased ALDH1L1 is associated with a poor prognosis [44]. Not surprisingly, in our results, the ALDH1L1 was indicated as a potential poor prognostic biomarker for GC, and that the promoter methylation may be a major mechanism controlling ALDH1L1 levels in human cancers [45].

In *ONCOMINE* datasets, ALDH1A3 expressed lower in GC than in normal tissues. Although no researches illustrated significant correlations of ALDH1A2 or ALDH1L1 with GC in *ONCOMINE* datasets, high transcriptions of ALDH1A2, ALDH1A3 and ALDH1L1 isoenzymes were found to be correlated with shorter OS in all GC, no matter the type of GC or HER2 status in the patients. GC affect more male than female, so we analyzed the survival in different gender (Supplementary Figure S2) and found that these results were independent of gender, supporting that ALDH1A2, ALDH1A3 and ALDH1L1 were the main contributors for ALDH1 activities in GC and potential prognostic markers and treated targets for GC. The results provide better understanding of the heterogeneity and complexity in the molecular biology of GC, which is the basement of more accurate prognosis and development of new treatment targets.

## MATERIALS AND METHODS

### ONCOMINE analysis

The mRNA levels of different *ALDH1* isoforms in different cancers were analyzed by *ONCOMINE* gene expression array datasets (www.oncomine.org), which is an online cancer microarray database to facilitate discovery from genome-wide expression analyses [46]. In this study, we compared the clinical specimens of cancer vs. normal control datasets, using a Students' *t*-test to generate a *p*-value. The *p* value was set up at 0.01 and fold change was defined as 2, whereas the data type was restricted to mRNA. Significant correlations can be found in different researches of GC, showed in typical figures.

### The kaplan-meier plotter

The prognostic value of the mRNA expression of ALDH1 isoforms in GC was assessed using the Kaplan-Meier plotter (www.kmplot.com), which is also an online database, including gene expression data and clinical data [47]. Up to now, Kaplan-Meier plotter is capable to assess the effect of 54, 675/22, 277 genes on survival of 10, 188 clinical cancer samples, including 4, 142 breast, 1, 648 ovarian, 2, 437 lung and 1, 065 gastric cancer patients. The patient samples were divided into two groups, according to the expression of ALDH1 isoforms (high vs. low expression). We analyzed the overall survival of the patients with GC using a Kaplan-Meier survival plot. The JetSet best probe set of 5 ALDH1 sub-members (ALDH1A1, ALDH1A2, ALDH1A3, ALDH1B1, and ALDH1L1) were entered into the database (http://kmplot.com/analysis/index.php? p = service and cancer = gastric) obtain Kaplan-Meier plots in which the number-at-risk is indicated below the main plot. The hazard ratio (HR) with 95% confidence intervals and log rank *p* value was calculated and displayed on the webpage. HER2 status was determined using the gene chip probe set 216836_s_at as described before [48].

## SUPPLEMENTARY MATERIALS FIGURES


