# Supplementary material for: Mining distinct aldehyde dehydrogenase 1 (ALDH1) isoenzymes in gastric cancer
Source: Oncotarget. 2016 Mar 23;7(18):25340–9. doi: 10.18632/oncotarget.8294 (PMC5041908; doi:10.18632/oncotarget.8294)
Supplement: Supplementary file 1 [file oncotarget-07-25340-s001.pdf]

Mining distinct aldehyde dehydrogenase 1 (ALDH1) isoenzymes in gastric cancer

Supplementary Materials

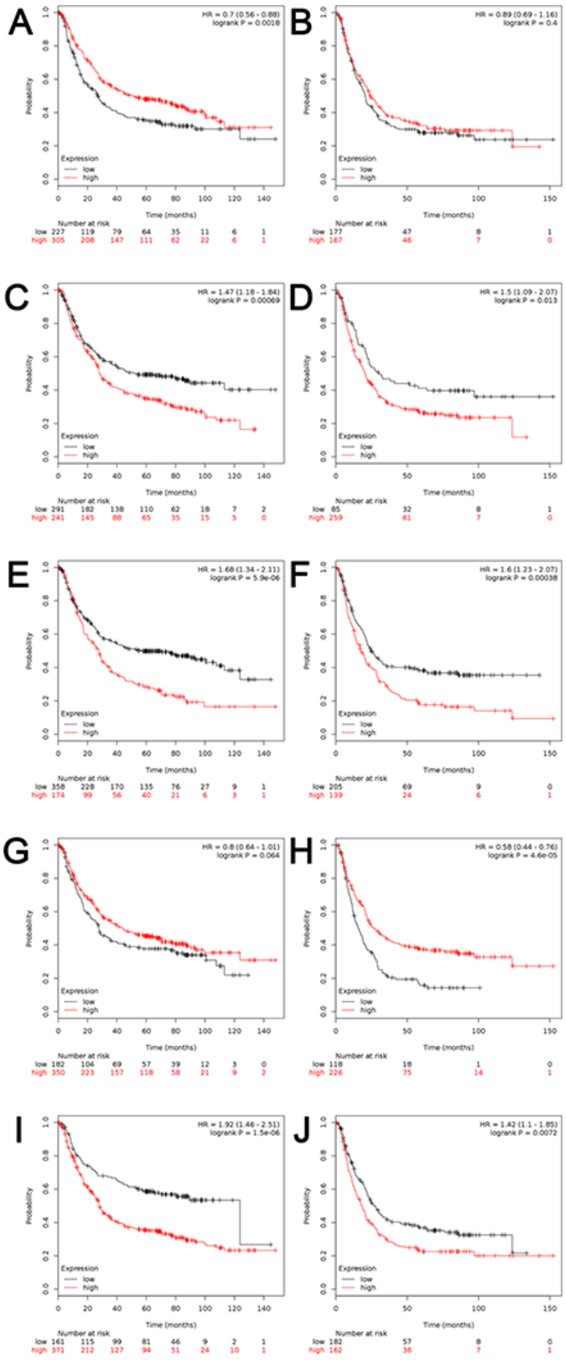

Supplementary Figure S1: The prognostic value of mRNA level of ALDH1 isoenzymes in HER2-negative (*n* = 532) and HER2-positive (*n* = 344) GC patients. (**A**, **B**) ALDH1A1 (212224_st) (**C**, **D**) ALDH1A2 (207016_s_at). (**E**, **F**) ALDH1A3 (203180_at). (**G**, **H**) ALDH1B1 (209646_×_at). (**I**, **J**) ALDH1L1 (205208_at). (A, C, E, G, I) Survival curves in HER2-negative GC patients (*n* = 532). (B, D, F, H, J) Survival curves in HER2-positive GC patients (*n* = 344). Data was analyzed using Kaplan-Meier plotter.

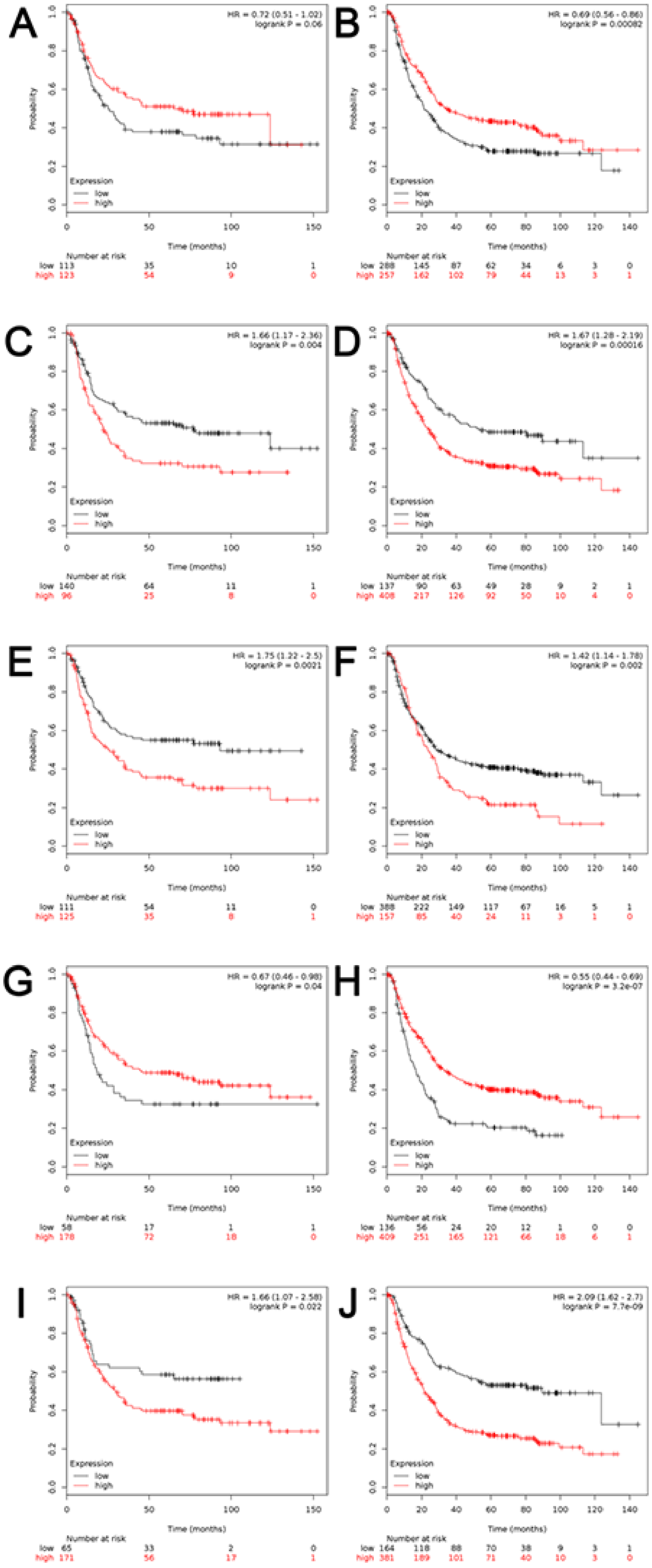

Supplementary Figure S2: The prognostic value of mRNA level of ALDH1 isoenzymes in female (*n* = 244) and male GC (*n* = 567) patients. (**A**, **B**) ALDH1A1 (212224_st) (**C**, **D**) ALDH1A2 (207016_s_at). (**E**, **F**) ALDH1A3 (203180_at). (G, H) ALDH1B1 (209646_×_at). (**I**, **J**) ALDH1L1 (205208_at). (A, C, E, G, I) Survival curves in female GC patients (*n* = 244). (B, D, F, H, J) Survival curves in male GC patients (*n* = 567). Data was analyzed using Kaplan-Meier plotter.
